# Increased frequency of porcine epidemic diarrhea virus shedding and lesions in suckling pigs compared to nursery pigs and protective immunity in nursery pigs after homologous re-challenge

**DOI:** 10.1186/s13567-016-0402-5

**Published:** 2016-11-21

**Authors:** Priscilla F. Gerber, Chao-Ting Xiao, Kelly Lager, Kimberly Crawford, Vikas Kulshreshtha, Dianjun Cao, Xiang-Jin Meng, Tanja Opriessnig

**Affiliations:** 1 The Roslin Institute and the Royal (Dick) School of Veterinary Studies, University of Edinburgh, Midlothian, Scotland, UK; 2 Department of Veterinary Diagnostic and Production Animal Medicine, Iowa State University, Ames, IA USA; 3 College of Biology, Hunan University, Changsha, China; 4 National Animal Disease Center, United States Department of Agriculture-Agricultural Research Services, Ames, IA USA; 5 Department of Biomedical Sciences and Pathobiology, College of Veterinary Medicine, Virginia Polytechnic Institute and State University, Blacksburg, VA USA

## Abstract

Porcine epidemic diarrhea virus (PEDV) causes enteric disease in pigs and spreads rapidly after entering naïve pig populations. The objectives were to (1) compare the disease course following inoculation with PEDV isolate US/Colorado/2013 in naïve 10 day and 8 week-old pigs, and (2) contrast the naïve response to homologous challenge in 8 week-old pigs. Pigs were randomly assigned into group 1 (*n* = 40, no PEDV exposure), group 2 (*n* = 43, PEDV inoculation at 10 days of age) and group 3 (*n* = 48, PEDV inoculation at 8 weeks of age). Thirty-three group 2 pigs received a homologous challenge at 8 weeks of age. Following primary or secondary inoculation, 3–10 pigs were euthanized at days post-inoculation (dpi) 1, 2, 3, 7 or 14. Clinical signs were more pronounced in 10 day-old pigs compared to 8 week-old pigs at dpi 2 and 3, a higher number of 10 day-old pigs shed PEDV RNA in feces compared to 8 week-old pigs. Typical severe atrophic enteritis of PEDV infection was observed at dpi 3 in both age groups, and at dpi 4 and 14 fecal shedding patterns were also similar. While both age groups had seroconverted to PEDV by dpi 14, IgG levels were higher in 8 week-old pigs. PEDV IgA antibodies were detected in feces of approximately 50% of the pigs at dpi 44. In homologous challenged pigs, no clinical signs or lesions were found, and PEDV fecal shedding was restricted to less than 10% of the pigs indicating the existence of homologous protection 44 days after initial PEDV exposure.

## Introduction

Porcine epidemic diarrhea virus (PEDV), a member of the genus *Alphacoronavirus* in the family *Coronaviridae*, is highly contagious and causes enteric disease characterized by acute vomiting and diarrhea in pigs of all ages [1]. PEDV infection has been reported in swine producing countries in Europe, Asia, the Americas and the Caribbean [2]. Based on amino acid differences in the N-terminal domain of the spike (S) gene, PEDV can be grouped into genogroups (G) 1 and 2, which are further subdivided into G1a, G1b, G2a and G2b [3, 4]. Most PEDV strains circulating in Europe and Asia prior to 2010 belong to G1a, including classical strains such as CV777 [5]. Panzootic strains of PEDV G2b, also known as non-INDEL strains, have been associated with large scale outbreaks with high rates of illness and death in naïve suckling piglets worldwide while PEDV G2a is restricted to Asia [2]. Genogroup 1b strains, also known as INDEL strains, have been reported in Asia, North America and Europe and comprise variant strains that contain genetic signatures of the classical G1a strains in their S gene [4].

Age-resistance to disease induced by PEDV infection has been reported and neonatal (1–9 day-old) piglets often display more severe clinical signs than weaned (3–4 week-old) pigs [6–11]. PEDV infection in neonatal piglets has been described to produce earlier onset of viral shedding and more severe clinical disease when compared to older pigs [6, 9–11]. Experimental infection of 8-12 week old pigs has been shown to produce either no clinical signs [6] or only mild diarrhea that lasted for several days [12], while 4 week-old pigs were more clinically affected when receiving the same PEDV inoculum dose [6, 12]. Although the innate and adaptive immune responses and pathogenesis of PEDV infection for neonatal and weaned pigs have been described [7–11], little is known on pathological lesions and mucosal immune responses following PEDV infection and re-challenge in older pigs.

Protective immunity to homologous G1a or G2b PEDV challenge has been observed in pigs challenged 3–7 weeks after the first infection [12, 13]. In addition, sows naturally infected with PEDV G1b seem to be resistant to experimental infection with PEDV G2b up to 7 months after initial exposure [14]. However, experimental infection of 3–4 day-old pigs with a G1b PEDV strain followed by a G2b PEDV challenge 21–29 days post-inoculation (dpi) induced only partial cross-protection [15]. Protection from G1a PEDV homologous challenge 3 weeks after first infection was correlated with the number of IgG and IgA antibody secretory cells present in the gut-associated lymphoid tissues and blood but not in the spleen in weaned pigs [16]. However, there is limited information on mucosal immunity after PEDV infection in older pigs [17]. The objectives of this study were to investigate the clinical disease course including clinical signs, viral shedding in feces, lesions and humoral immune responses in 10 day-old pigs compared to 8 week-old pigs after primary PEDV infection and in 8 week-old pigs with primary PEDV infection compared to pigs with homologous challenge after initial PEDV infection at 10 days of age.

## Materials and methods

### Animals and experimental design

One-hundred-thirty-one PEDV-negative crossbred piglets farrowed at the National Animal Disease Center (NADC), USDA-ARS Ames from gilts bred at NADC were weaned at 6–7 days of age. Initially, the weaned piglets were fed a commercial reconstituted milk supplement multiple times per day and a commercial pelleted starter ration ad lib. After approximately 7 days, the frequency of milk feedings per day was reduced and discontinued at approximately 14 days after weaning. At approximately 21 days after weaning the pigs were transitioned to a custom prepared pelleted ration and limit fed for the duration of the study. At weaning, the pigs were randomly divided into the following treatment groups and housed in separate biosecurity level 2 isolation rooms: Group 1 (*n* = 40, no PEDV exposure), group 2 (*n* = 43, PEDV inoculation at 10 days of age) and group 3 (*n* = 48, PEDV inoculation at 8 weeks of age) (Table 1). A subset of the group 2 pigs (*n* = 33) was inoculated a second time with PEDV at 8 weeks of age. Five to 30 pigs in each group were euthanized at dpi 3 or 14 (Table 1). In addition, a subset of 10 day-old pigs was necropsied at dpi 7, and subsets of group 3 pigs were necropsied at dpi 1 and 2 (Table 1). Blood samples were collected in serum separator tubes (Fisher Scientific, Pittsburgh, Pennsylvania, USA) at dpi 0, 7, 14 and 24 after first inoculation, and at dpi 0 and 14 after second inoculation. Serum was harvested following centrifugation at 3000 × *g* for 10 min at 4 °C, and tested for anti-PEDV IgA and IgG antibodies by enzyme-linked immunosorbent assay (ELISA). Polyester swabs were used to collect fecal samples daily from 0–24, 32, and 33 days after first inoculation, and from 0–3 and at 14 days after second inoculation. Swabs were stored at −80 °C in 5 mL plastic tubes containing 1 mL of sterile saline solution and tested for PEDV RNA by quantitative reverse transcriptase (RT)-PCR. Feces were collected on 0, 3 and 14 days after second inoculation and tested for anti-PEDV IgA antibodies by ELISA. A diarrhea score was recorded for each pig at dpi 0–7, 10, 12, 14, 17, 18, 21, 24, and 33 after first inoculation, and at dpi 0–4 after second inoculation. The score ranged from 0 to 3 and included 0 = normal, 1 = moist, 2 = pasty, and 3 = watery. Frequency of diarrhea was calculated by adding all days for a pig with a score of 2 or greater. Pigs were weighed at dpi 0 and 21.

**Table 1:** **Experimental design and time line**

Pig age	10 days			8 weeks				10 weeks
Group	Inoculation 1	N1	N2	Inoculation 2	N3	N4	N5	N6
	dpi 0/−44^a^	dpi 3/−41	dpi 7/−37	dpi 44/0	dpi 45/1	dpi 46/2	dpi 47/3	dpi 58/14
1 (*n* = 40)	Mock	10^b^	10	Mock	0	0	10	10
2 (*n* = 43)	PEDV	5	5	PEDV	0	0	10	23
3 (*n* = 48)	Mock	0	0	PEDV	5	3	20	20

^a^dpi related to inoculation 1/dpi related to inoculation 2.

^b^The number of pigs necropsied on a certain day.

### PEDV inoculation

For PEDV inoculations, the stock of G2b PEDV strain US/Colorado/2013 (CO-13) was passaged three times to a titer of 9 × 10^4^ plaque-forming unit (PFU) per mL, which was used for inoculation. Near complete sequencing of the inoculum stock indicated 99.5% identity with the parental strain KF272920 (data not shown). Each pig in group 2 (at 10 days of age) and 3 (at 8 weeks of age) received 10 mL of the inoculum stock orally by slowly dripping the inoculum into the oral cavity of the pig (Table 1). The negative control pigs were inoculated orally in a similar fashion with 10 mL of virus free cell culture medium.

### Antibody detection

Serum samples were tested for the presence of anti-PEDV IgG and IgA antibodies by  in-house PEDV S protein 1 (S1)-based indirect ELISAs [18, 19]. Fecal samples were tested for anti-PEDV IgA antibodies by ELISA as described [19]. For the IgG detection in serum, the sample to positive (S/P) ratios of >0.2 were considered antibody positive, between 0.14 and 0.2 as suspect, and <0.14 as negative. For the IgA ELISA in serum and fecal samples an S/P ratio less than 0.14 was considered negative and an S/P ratio higher or equal to 0.14 was considered positive.

### RNA extraction and quantitative real-time RT-PCR for PEDV

Fecal swab suspensions were tested for the presence of PEDV RNA by quantitative RT-PCR assay using primers and probe combination as previously described [20]. To confirm that the PEDV from the experimentally infected pigs was the same as the virus in the inoculum, the S1 gene was amplified and sequenced [20].

### Necropsy

All pigs were euthanized with pentobarbital (Fatal-plus^®^, Vortech Pharmaceuticals, LTD, Dearborn, MI, USA) and necropsied. Nine sections of small intestines (duodenum, jejunum, ileum) and three sections of large intestines (colon) were collected at necropsy, fixed in 10% neutral-buffered formalin, and routinely processed for histological examination.

### Histopathology and PEDV immunohistochemistry (IHC)

Microscopic lesions were evaluated by a veterinary pathologist blinded to the treatment status. Sections of small and large intestines were evaluated for the presence of inflammation, villus atrophy and necrosis based on the following criteria: 0 = normal section with intact epithelium and villi, 1 = mild diffuse inflammatory cell infiltrate in mucosa and submucosa and/or multifocal mild villous blunting, 2 = moderate inflammatory cell infiltrates in mucosa and submucosa and/or moderate villous blunting, 3 = marked mucosal, submucosal and transmural inflammatory cell infiltration and/or diffuse severe villous atrophy. PEDV-specific antigen was detected by IHC on selected formalin-fixed and paraffin-embedded sections of intestinal sections using monoclonal antibody specific for PEDV (BioNote, Hwaseong-si, Gyeonggi-do, Korea) [21, 22]. The amount of PEDV antigen was scored by a pathologist blinded to treatment status based on the following criteria: 0 = no signal, 1 = 1–10% of villous enterocytes within the section showing a positive signal, 2 = 11–50% of villous enterocytes showing a positive signal, and 3 = more than 50% of villous enterocytes showing a positive signal.

### Statistical analysis

Summary statistics were calculated for groups. Fisher’s exact test was applied to compare the proportions of positive and negative results for PEDV shedding, presence or absence of diarrhea and presence or absence of lesions between groups in different experiment days. PEDV shedding patterns overtime were analyzed and compared between pigs infected at 10 days of age (group 2) or 8 weeks of age (group 3) by repeated measures analysis of variance (ANOVA), and the antibody levels for these groups were analyzed by ANOVA. Differences among the interacting groups were assessed using Tukey’s t-test. A *P* value of less than 0.05 was considered significant. Non-parametric Kruskal–Wallis ANOVA was used on non-repeated data (histopathological and IHC scoring), and Mann–Whitney tests were used to evaluate differences between pairs. Analyses were performed using a commercial statistical software (Minitab 17, State College, PA, USA).

## Results

### Non-infected pigs

The non-infected control pigs did not develop clinical signs or lesions and remained free of PEDV RNA and antibodies for the duration of the study.

### Comparison of pigs infected with PEDV at 10 days or at 8 weeks of age

#### Clinical signs

Other than diarrhea, no other clinical signs associated with PEDV infection were observed. The percentage of affected pigs and their mean diarrhea score are summarized in Table 2. The 10 day-old pigs had mild diarrhea starting at dpi 1 (4.6%, 2/43) while 8 week-old pigs had mild diarrhea starting at dpi 2 (7.5%, 3/40). Although there were no significant differences in severity of diarrhea in 10 day-old pigs compared to 8 week-old pigs following PEDV inoculation, there was a trend for an earlier onset and a higher diarrhea score in younger pigs (Table 2).

**Table 2:** **Percentage of pigs with diarrhea and percentage of PEDV RNA positive rectal swabs**

dpi	Percentage of pigs with diarrhea (positive/total number animals, median fecal consistence of pigs with diarrhea)		Percentage of PEDV RNA positive rectal swabs (positive/total number animals, mean log genomic copies per positive rectal swab ± SD)	
	10 day-old pigs	8 week-old pigs	10 day-old pigs	8 week-old pigs
1	4.6%^A,1^ (2/43, 2)	0/43^A^	18.6%^a,2^ (8/43, 4.9 ± 1.7)	18.7^a^ (9/48, 5.4 ± 1.3)
2	18.6%^A^ (8/43, 2)	7.5%^A^ (3/40, 2)	97.6%^a^ (42/43, 5.7 ± 1.6)	74.4^b^ (32/43, 6.1 ± 2.1)
3	23.2%^A^ (10/43, 3)	6.6%^B^ (2/30, 2.5)	100%^a^ (43/43, 7.1 ± 0.8)	88.3^b^ (38/43, 6.6 ± 1.9)
4	18.6%^A^ (13/38, 2)	13.3%^B^ (4/30, 2.5)	100%^a^ (38/38, 7.1 ± 1.0)	100^a^ (20/20, 7.5 ± 0.9)
14	0/33^A^	0/20^A^	60.6%^a^ (20/33, 4.1 ± 1.1)	45.0^a^ (9/20, 3.6 ± 0.7)

^1^Different uppercase font superscripts ^(A,B)^ indicate differences (*P* = 0.05) in the number of positive animals in the two age groups on a certain dpi.

^2^Different regular font superscripts ^(a,b)^ indicate differences (*P* < 0.05) in the number of positive animals in the two age groups on a certain dpi. There was no difference between the mean log_10_ genomic copies per positive rectal swabs between age groups.

#### PEDV shedding in feces

**Figure 1:**
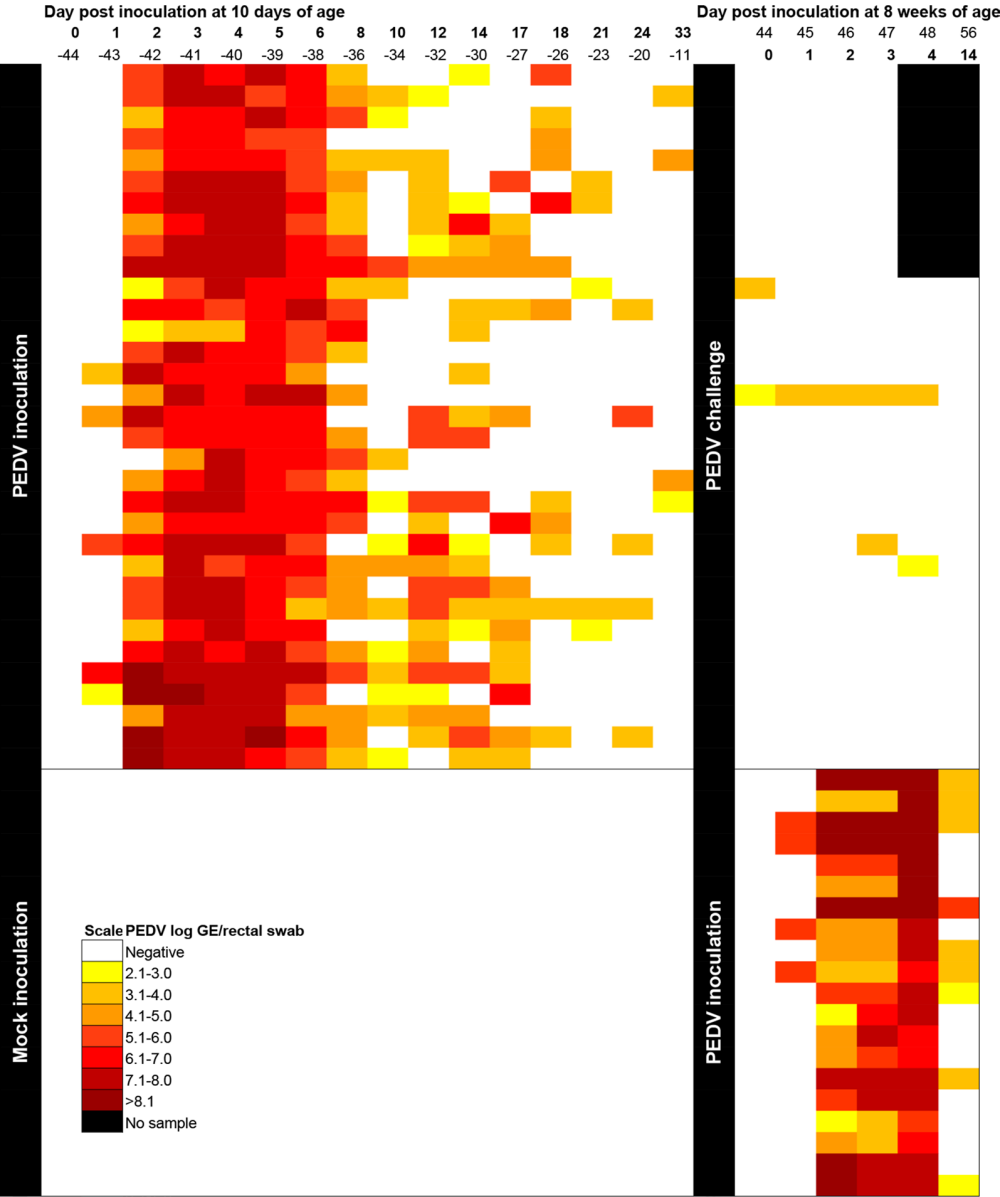
**PEDV RNA fecal shedding.** Fecal swabs were collected over time from 33 pigs infected with PEDV at 10 days of age and challenged at 8 weeks of age and from 20 pigs infected with PEDV at 8 weeks of age. The quantity of virus shed for each pig for each day is shown with different color intensity.

Percentage of pigs shedding PEDV in rectal swabs and the mean genomic copies per swab from dpi 0-4 and dpi 14 are summarized in Table 2. PEDV RNA was detected in rectal swabs from dpi 1 through 14 in 10 day-old and 8 week-old infected pigs (Figure 1; Table 2). By dpi 1, the frequency of shedding PEDV RNA in rectal swabs and the viral loads were similar (*P* > 0.05, Table 2) in 10 day-old pigs (18.6%, 8/43) and 8 week-old pigs (18.7%, 9/48). Although lower numbers of 8 week-old pigs shed PEDV at dpi 2 and 3 compared to 10 day-old pigs, RT-PCR positive pigs shed a similar amount of virus in both age groups (*P* < 0.05, Table 2). Sequencing of the PEDV S1 region of selected PEDV RNA positive samples from each PEDV-infected group confirmed 100% sequence identity to the strain used for inoculation.

#### IgG antibody levels

**Figure 2:**
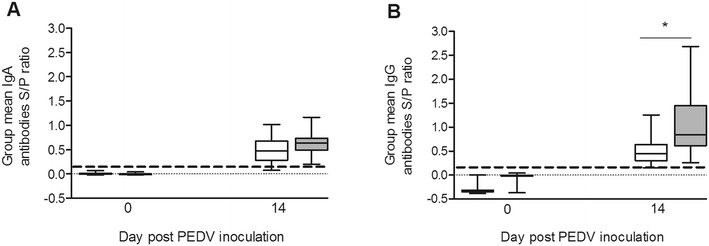
**Anti-PEDV IgA (A) and IgG (B) antibodies levels in serum of PEDV infected pigs.** Serum samples were collected from PEDV infected pigs at dpi 0 and dpi 14. Pigs infected with PEDV at 10 days of age are indicated by white bars and pigs infected with PEDV at 8 weeks of age are indicated by grey bars. Data are presented as mean ELISA S/P ratios. The asterisk indicates a significant difference in group mean S/P ratios.

By dpi 14, the 33 remaining 10 day-old pigs and the 20 remaining 8 week-old pigs had seroconverted to PEDV (Figure 2). The IgG antibody levels in 8 week-old pigs were higher compared to 10 day-old pigs (Figure 2).

#### IgA antibody levels

By dpi 14, 93.9% (31/33) of the 10 day-old pigs had detectable IgA antibodies against PEDV while 100% (20/20) of the 8 week-old pigs had IgA antibodies against PEDV in serum (Figure 2). There was no difference between the IgA levels between age groups.

#### Microscopic lesions and PEDV antigen in tissues

**Figure 3:**
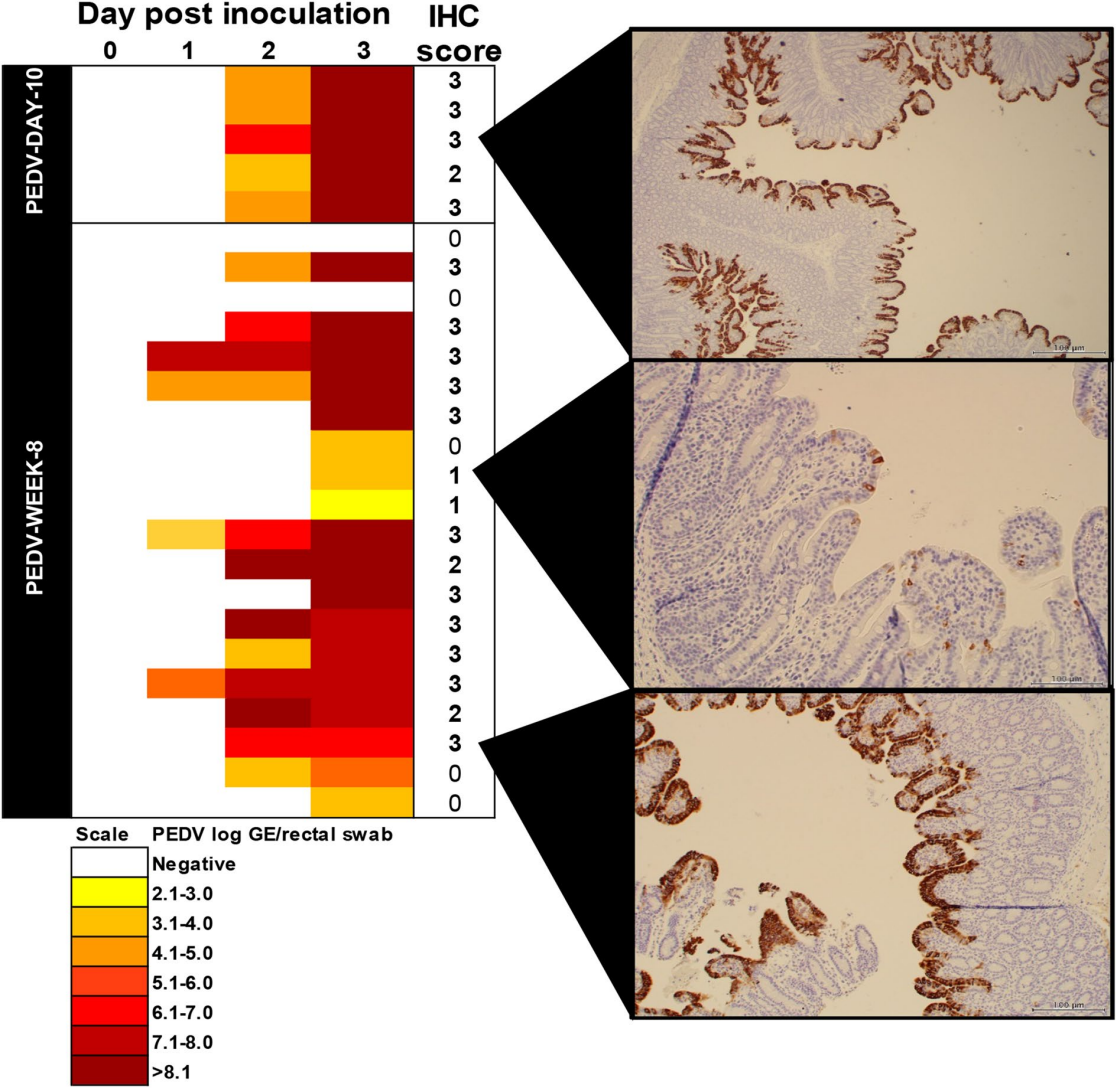
**PEDV RNA fecal shedding and intralesional antigen levels in PEDV infected pigs.** Fecal swabs to determine PEDV shedding were collected on dpi 1, 2 and 3 and PEDV IHC scores to determine intralesional antigen in intestinal sections were obtained at dpi 3 from pigs infected with PEDV at 10 days or at 8 weeks of age. IHC scores range from 0 = negative to 3 = abundant amount of PEDV antigen. The quantity of virus shed for each pig for each day is shown with different color intensity.

At the peak of diarrhea at dpi 3, all five 10 day-old pigs had severe diffuse villus atrophy throughout the small intestines (mean score: 2.2) with multifocal low numbers of lymphocytes and neutrophils and foci of necrosis associated with abundant PEDV antigen in small intestinal sections (mean score 2.1; Figure 3). In addition, 2 of the 5 pigs also had mild suppurative colitis (score 1) associated with PEDV antigen (mean score 0.3). While viral enteritis was still detectable at dpi 7 and manifested as mild-to-moderate villus atrophy in all 10 day-old pigs (mean score 1.6), the amount of PEDV antigen was decreasing (3/5 pigs; mean score 1). Microscopic lesions for 8 week-old pigs are summarized in Table 3. Similar to 10 day-old pigs, by dpi 3 13/20 pigs had moderate-to-severe atrophic enteritis throughout the small intestines associated with abundant amounts of PEDV antigen (Figure 3; Table 3). By dpi 14, lesions in 8 week-old pigs had completely resolved (Table 3).

**Table 3:** **Severity of microscopic lesions and presence of PEDV antigen determined by IHC in tissues from pigs infected with PEDV at 8 weeks of age**

Segment^a^	dpi 1		dpi 2		dpi 3		dpi 14	
	Lesions^b^	PEDV^c^	Lesions	PEDV	Lesions	PEDV	Lesions	PEDV
Duodenum	0/5	0/5	0/3	1/3 (3, 1)^d^	13/20 (2, 1)	15/20 (3, 2.3)	0/30	0/30
Ileum	0/5	0/5	0/3	1/3 (3, 1)	13/20 (3, 2)	15/20 (3, 3)	0/30	0/30
Jejunum	0/5	0/5	0/3	1/3 (1, 0.3)	13/20 (3, 3)	15/20 (3, 3)	0/30	0/30
Colon	0/5	0/5	0/3	0	0/20	6/20 (1, 0.3)	0/30	0/30
Mesenteric lymph node	0/5	0/5	0/3	0/3	0/20	12/20 (2, 1)	0/30	0/30

^a^Three sections were collected and evaluated for each intestinal segment.

^b^Microscopic lesions included villous atrophy and lymphocytic inflammation and were scored as 0 = absent to 3 = severe.

^c^Prevalence and amount of PEDV antigen were scored as 0 = absent to 3 = abundant for each intestinal segment.

^d^The average score of three sections of each segment was calculated as the segment score. Data presented as prevalence (highest score in a segment, median segment score).

### Pigs infected with PEDV at 10 days and challenged at 8 weeks of age

#### Course of the PEDV infection in 10 day-old pigs until challenge

**Figure 4:**
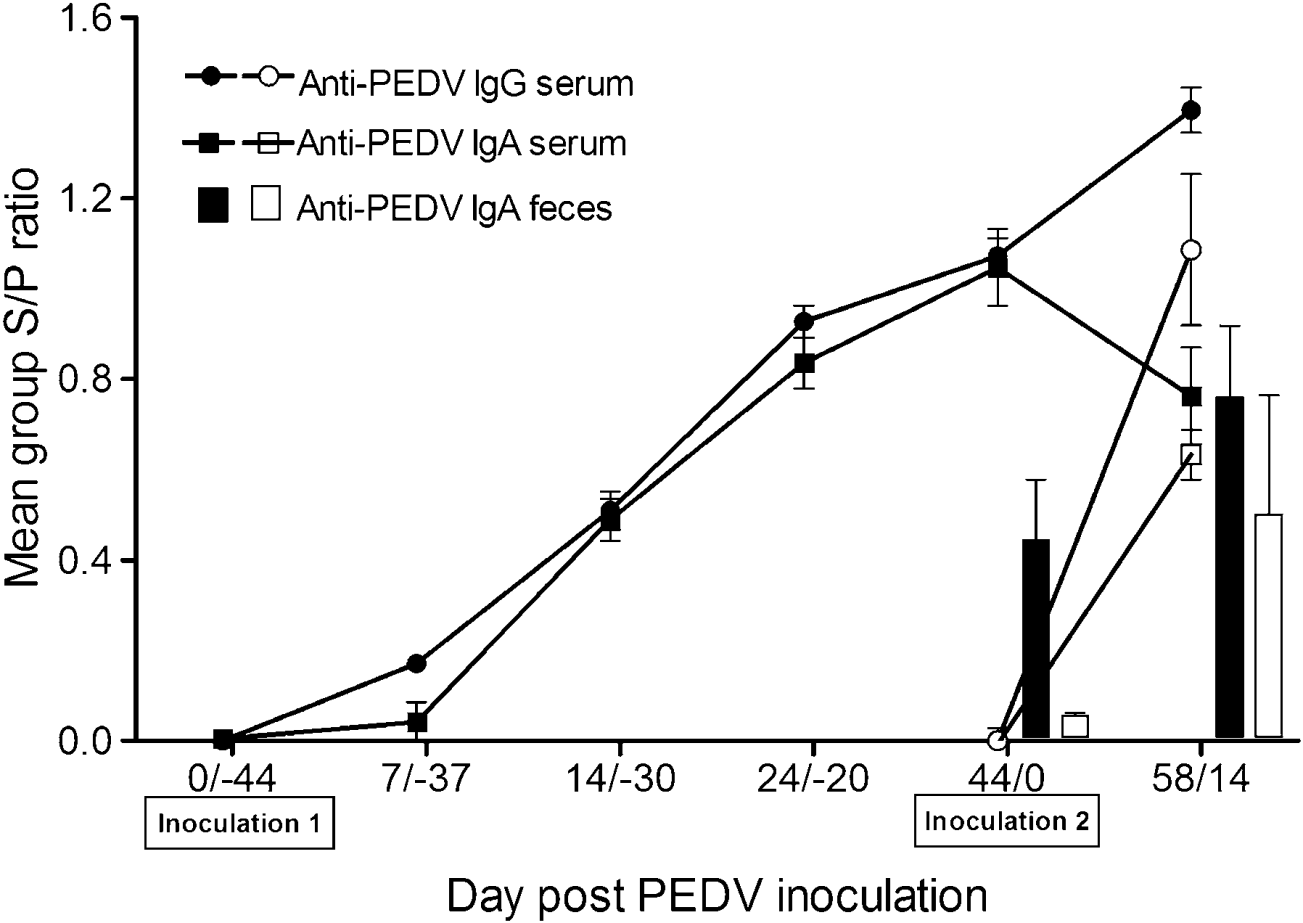
**Anti-PEDV IgA (serum and feces) and IgG (serum) levels in PEDV infected or challenged pigs.** Pigs infected with PEDV at 10 days of age and challenged at 8 weeks (Group 2, black circles, squares or bars) were compared to pigs infected with PEDV at 8 weeks of age (Group 3, white circles, squares or bars). Inoculation 1 indicates infection at 10 days of age and inoculation 2 indicates challenge at 8 weeks of age. Feces were only collected at inoculation 2 (dpi 44/0) and two weeks later (dpi 58/14).

The peak of viral shedding in rectal swabs preceded the peak of diarrhea by two days in 10 day-old pigs. Pigs started to shed virus at dpi 1/−43 to 3/−41, and continued for 5–13 days (median 6) and then intermittently shed virus until dpi 33/−11 (Figure 1). The peak of PEDV shedding occurred between dpi 3/−41 and dpi 6/−38 with 100% of the 10 day-old pigs shedding PEDV RNA (Figure 1) followed by the peak of diarrhea at dpi 5/−39 when 84% (32/38) of the pigs had moderate watery diarrhea (positive fecal consistence score range 2–3, median 3). PEDV RNA shedding started to decrease at dpi 8/−36 when 81.8% (27/33) of the pigs shed PEDV RNA and was linked with decrease of diarrhea to 6.1% (2/33) between dpi 7/−37 and dpi 12/−32. Fecal composition returned to normal standards around dpi 22/−22 (positive fecal consistence score 1, median 0). The weight gain during the first 3 weeks after PEDV infection was not different for 10 day-old pigs infected with PEDV compared to non-infected control pigs (data not shown). Seroconversion for PEDV started by dpi 7/−37, when 42% (16/38) of the pigs were positive for IgG antibodies against PEDV and 10.5% (4/38) of the pigs were positive for IgA antibodies against PEDV (Figure 4).

#### Course of initial PEDV infection or PEDV challenge in 8 week-old pigs

All pigs that had been infected with PEDV at 10 days of age and were free of clinical disease at the time of reinfection. All challenged pigs were positive for serum IgG and IgA PEDV antibodies at 8 weeks of age, and 51.5% (17/33) had detectable IgA anti-PEDV antibodies in fecal samples (Figure 4). Clinical signs were not observed after challenge whereas pigs with initial infection at 8 weeks of age had diarrhea for several days (Table 2). At reinfection, 6.1% (2/33) of the challenged pigs shed PEDV RNA (maximum of 3.2 log_10_ PEDV RNA/rectal swab) (Figure 1) and 9% (3/33) shed low levels of PEDV RNA (maximum of 3.6 log_10_ PEDV RNA) from dpi 1 to 4 (Figure 1). Detailed results for pigs with initial infection at 8 weeks of age are summarized in Table 2. In general, 74.4–100% of the 8 week-old pigs shed high PEDV RNA levels (maximum of 9.1 log_10_ PEDV RNA, median of positives 7.1) from dpi 2 to 4. PEDV associated microscopic lesions or PEDV antigen were not detected in any of the 10 challenged pigs at dpi 3 (data not shown) whereas most (13/20) pigs with initial PEDV infection at 8 weeks of age developed visible microscopic lesions (*P* < 0.01) (Table 3). In challenged pigs IgG antibody levels in serum increased over the following 14 days (dpi 58 post initial PEDV infection) to 1.4 ± 0.1 (Figure 4). By dpi 14, 86.9% (20/23) of the challenged pigs also had detectable IgA antibodies in feces (Figure 4). Interestingly, the mean S/P IgA anti-PEDV antibody ratios decreased in serum samples while it increased in fecal samples after the challenge (Figure 4). By dpi 14, the levels of IgG and IgA in serum and IgA levels in feces of the 8 week-old challenged pigs were similar to those of 8 week-old pigs infected for the first time (*P* > 0.05) (Figure 4).

## Discussion

It has been demonstrated that enteric clinical signs and viral shedding after PEDV infection are more severe in neonatal pigs when compared to weaned pigs [9, 10]. However, histological and immunological information about the apparent age-resistance to PEDV infection in older pigs was lacking. In the present study, fecal shedding started at dpi 1 for the pigs infected at 10 days of age and the pigs infected at 8 weeks of age. This is in contrast to other studies that describe a delay of one day in onset of fecal shedding in weaned pigs compared to neonatal pigs [9, 10]. Differences in the PEDV strains, RT-PCR assays and the higher number of pigs used in the present study may partially explain the differences found. Although less 8 week-old pigs shed virus at dpi 2 and 3, the virus load detected in positive animals was similar to that in pigs infected at 10 days for the duration of the study. A similar trend was previously reported in 9 day-old pigs and 26 day-old pigs infected with US G2b PEDV strain PC21A [10], although another study reported a higher shedding of PEDV for 5 day-old pigs when compared to 3 week-old pigs infected with US G2b PEDV strain US/IN19338/2013 [9].

Pigs infected at 10 days of age had higher frequency of diarrhea compared to pigs infected at 8 weeks of age. Among the 10 day-old pigs, up to 84% of the pigs suffered from diarrhea for several days starting at dpi 1; while among 8 week-old pigs, up to 13% had mild to moderate diarrhea starting at dpi 2. Previous work has shown that 11 week-old pigs infected for the first time developed intermittent mild diarrhea beginning by dpi 2–3 that lasted for several days with PEDV RNA shedding in feces after dpi 2 [12]. Despite differences in the clinical presentation at dpi 3 there was no difference in the degree of microscopic lesions and amount of PEDV antigen in the intestines of affected 10 day-old pigs or 8 week-old pigs. This finding is similar to what has been found previously for 9 day-old pigs and 26 day-old pigs [10]. Age-dependent differences in this study included prevalence rates of pigs that developed recognizable microscopic lesion, 100% in 10 day-old pigs but only 65% in 8 week-old pigs. If pigs developed lesions, the severity of the lesions was associated with the levels of PEDV antigen in both age groups.

Protection after homologous challenge at 11 week of age, 7 weeks after initial PEDV exposure at 4 weeks of age, has been described [12]. However, microscopic lesions and IgA immunity have not been assessed. Similar to the previous study, 8 week-old pigs in this study were protected when receiving a homologous PEDV challenge 7 weeks after initial PEDV inoculation at 10 days of age based on the absence of clinical signs, viral shedding in feces, and microscopic lesions and PEDV antigen in tissue sections. Challenged pigs had a clear anamnestic response based on serum IgG levels and fecal IgA levels after challenge, while there was a decline of IgA levels in serum samples. Previously, IgA antibodies profiles in serum have been demonstrated in 11 day-old pigs and 3 week-old pigs experimentally infected with PEDV strain CV777 [23]. In that study, at dpi 21 serum IgA and IgG antibody levels correlated with protection against homologous PEDV challenge [23]. In addition, the numbers of IgA and IgG antibody-secreting cells in blood samples collected at challenge also correlated with the responses identified in the gut associated lymphoid tissues [23].

Although antibody-secreting cells were not evaluated in this study, more than 50% of the challenged pigs had detectable PEDV IgA antibodies in fecal samples at the time of the challenge indicating that local immunity is long lasting following PEDV infection. It has been reported that PEDV specific IgA antibodies in feces of naturally infected sows disappeared approximately 1–2 months after PEDV infection, despite the presence of PEDV-specific IgA and IgG antibody secreting cells in intestines and lymphoid tissues for at least 6 months and presence of IgA in serum [17]. Indeed, when evaluating a subset of 10 pigs in this study, the number of IgA positive pigs rose from 30% at reinfection to 70% 3 days later, showing a rapid secondary response after challenge (data not shown).

PEDV clinical signs were more severe and viral shedding occurred at higher rates during acute infection in 10 day-old pigs but microscopic lesions and overall amount of viral shedding were similar compared to 8 week-old pigs and by dpi 14, 8 week-old pigs had higher levels of PEDV-specific IgG antibodies in serum. Local mucosal immunity measured by determining the presence of fecal PEDV IgA was detected 44 days after primary PEDV exposure in approximately 50% of the pigs and rapidly rose in pigs as soon as 3 days after homologous PEDV reinfection. Pigs had no clinical signs and only sporadic virus shedding after homologous PEDV challenge 44 days after initial exposure indicating protection.

## Abbreviations

ANOVA: analysis of variance
dpi: day post-inoculation
ELISA: enzyme-linked immunosorbent assay
G: genogroup
Ig: immunoglobulin
IHC: immunohistochemistry
N: necropsy
NADC: National Animal Disease Center
PCR: polymerase chain reaction
PEDV: porcine epidemic diarrhea virus
PFU: plaque forming unit
RNA: ribonucleic acid
RT: reverse transcriptase
S: spike
S/P ratio: sample-to-positive ratio
USDA-ARS: United States Department of Agriculture, Animal Research Service
